# Distribution of high and low risk HPV types by cytological status: a population based study from Italy

**DOI:** 10.1186/1750-9378-6-2

**Published:** 2011-01-20

**Authors:** Paolo Giorgi Rossi, Francesco Chini, Simonetta Bisanzi, Elena Burroni, Giuseppe Carillo, Amedeo Lattanzi, Claudio Angeloni, Aurora Scalisi, Rosalba Macis, Maria T Pini, Paola Capparucci, Gabriella Guasticchi, Francesca M Carozzi

**Affiliations:** 1 Laziosanità - Agency for Public Health, Lazio Region. Via di S. Costanza 53, 00198 - Rome, Italy; 2 Analytical and Biomolecular Cytology Unit, Cancer Prevention and Research Institute, ISPO, Via Cosimo il Vecchio 2, Florence - 50139, Italy; 3 Anatomia Patologica, Ospedale G. Moscati, ASL Caserta 2, Aversa (CE) - 81021, Italy; 4 Coordinamento Screening, ASL Teramo, Teramo - 64100, Italy; 5 Coordinamento Screening, ASL Catania, Catania - 95124, Italy; 6 Anatomia Patologica, ASL Cagliari 09125, Italy; 7 Coordinamento Screening, Dipartimento Materno Infantile, ASL Napoli 2, Giugliano in Campania (NA) - 80014, Italy; 8 Coordinamento Screening ASL Roma C, Rome, Italy

## Abstract

**Background:**

HPV type distribution by cytological status represents useful information to predict the impact of mass vaccination on screening programs.

**Methods:**

women aged from 25 to 64 who attended cervical cancer screening in five different Italian regions were tested for HPV infection with Hybrid Capture II (HCII) low and high risk probes. Women repeating Pap-test upon unsatisfactory or positive results, or as a post-treatment and post-colposcopy follow-up analysis, were excluded from our study. High risk (HR) HPV positive samples were typed using GP5+/GP6+ primed PCR, followed by Reverse Line Blot for 18 high/intermediate risk HPV types, while low risk (LR) HPV positive samples were tested with type specific primers for HPV6 and HPV11.

**Results:**

3410 women had a valid HCII and Pap-test. The prevalence of HR and LR infections was 7.0% and 3.6%, 29.1% and 13.7%, 68.1% and 31.9%, 60.0% and 0.0%, 65.0% and 12.0%, for negative, ASC-US, L-SIL, ASC-H and H-SIL cytology, respectively. The fraction of ASC-US+ cytology due to HPV 16 and 18 ranged from 11.2 (HPV 16/18 alone) to 15.4% (including HPV 16/18 in co-infection with other virus strains), and that due to HPV 6 and 11 ranged from 0.2% (HPV 6/11 alone) to 0.7% (including HPV 6/11 in co-infection with other LR virus strains).

**Conclusions:**

mass vaccination with bivalent or quadrivalent HPV vaccine would modestly impact on prevalence of abnormal Pap-test in screening.

## Background

In Italy, vaccination against HPV was included in the routine vaccination schedule at the beginning of 2008 and has since been actively offered to all 11-year old girls [1].

Since 2006, both the Ministry of Health and the Centre for Disease Prevention and Control have planned and commissioned a set of studies to assess HPV epidemiology before introducing the vaccine and to predict the impact of a mass vaccination campaign [2].

In particular, the aim of the present study was to measure the prevalence of different HPV types in the general population attending screening programs in the metropolitan area of Rome and southern Italy.

Here, we report the proportion of HPV types by cytological status. We calculated the proportion of abnormal cytology attributable to HPV 16-18 and 6-11 infection.

## Methods

### Setting

The study was conducted within cervical cancer screening programs that actively invite the entire target population (age ranging from 25 to 64) in Rome and southern Italy. A convenience sample of the existing screening programs was selected, because the study needed active participation to the local screening programs and formal approval from the Local Health Unit director. Nevertheless, our final sample included most of the well established programs in the south of Italy. The sample included 7 programs in 5 different regions: Abruzzo, Campania, Lazio, Sardinia, and Sicily. The planned sample size was 4000 women, 800 for each region. The structure and the statistical power of our sample have already been described in detail elsewhere [3]. The recruitment was conducted between December 2007 and September 2008.

### Sample collection

Cervical scrape samples were collected in ThinPrep vials containing PreservCyt (Cytyc Corp., Marlborough, USA) transport medium or in Specimens Transport Medium (STM) (DNAPAP cervical sampler, Qiagen, Gaithersburg, USA). Before testing, 400 μl of STM samples, vortexed on a shaking platform at 1000 rpm for at least 10 minutes, were biobanked at -80°C for typing procedures in case of HCII positivity. Only in one centre (Roma G., recruiting 400 women), the primary screening test was HPV, consequently data from this centre were not informative for this study.

### Cytological interpretation

The cytology was interpreted locally at each centre according to the Bethesda 2001 system (TBS2001) [4]. We grouped our results into four classes: negative (including benign cellular changes), ASC-US (including AGC), L-SIL, ASC-H, and H-SIL or more severe samples (including invasive squamous carcinoma, adenocarcinoma *in situ*). In two centres, pathologists reported also benign cellular changes or other reactive cellular abnormalities that refer to the previous classifications. In any case, these women were all referred to normal screening interval (i.e. three years) and were included in the negative cytology class.

All screening pathology units participate in routine quality control programs based on the circulation of standard slide sets [5].

### HPV testing and typing

The molecular biology methods have been described in detail elsewhere (3). Here we briefly report the adopted typing strategies.

The presence of high risk (HR) and low risk (LR) HPVs in cervical specimens was evaluated by Hybrid Capture II^® ^(HCII) (Qiagen, Gaithersburg, USA) (12, 13, 14, 15, 16, 17) using probemix B, specific for 13 HR HPV types: 16, 18, 31, 33, 35, 39, 45, 51, 52, 56, 58, 59 and 68, and probemix A, specific for 5 LR HPV types: 6, 11, 42, 43 and 44 [6]. The standard 1 pg/ml HPV DNA positivity threshold was adopted. HPV DNA testing was performed locally in 4 sites (one in Abruzzo, one in Campania and two in Rome) while samples collected in Cagliari and Catania were analysed in Florence (ISPO).

Typing procedures were centralized in Florence at the ISPO laboratory and all centres involved in the study sent HR or LR HPV positive samples there. The types included in IARC group 1 and 2a were classified as HR strains, the other types as LR ones [7].

DNA was extracted from 1.5 ml of samples in PreservCyt^® ^solution and 200 μl of STM samples using QIAamp DNAMini Kit.

HCII HR HPV positive specimens were amplified and typed with "consensus High Risk HPV genotyping kit" (Qiagen), a system based on PCR with biotinylated GP5+/GP6+ primers [8], followed by reverse Line Blot for 18 HR HPV types: 16, 18, 26, 31, 33, 35, 39, 45, 51, 52, 53, 56, 58, 59, 66, 68, 73 and 82.

GP5+/6+PCR-negative and reverse Line Blot-negative samples were amplified for the β-globin gene using GH20-PC04 primers (268 bp amplicon lenght) to assess the integrity of DNA [9].

β-globin positive samples were re-typed with "INNO-LiPA HPV genotyping Extra Amp" (Innogenetics, Ghant, Belgium) following the manufacturer's instructions. This system targets 28 HR or LR HPV types: 6, 11, 16, 18, 26, 31, 33, 35, 39, 40, 43, 44, 45, 51, 52, 53, 54, 56, 58, 59, 66, 68, 69, 70, 71, 73, 74 and 82. β-globin negative samples were extracted and typed again.

The remaining un-typed samples (GP5+/6+PCR-positive, reverse Line Blot-negative and INNO-LiPA negative) were considered untyped HPV (HPV X).

HCII LR HPV positive specimens were typed using HPV6 and HPV11 specific primers [10]. No LR type, other than 6 and 11, was searched for.

### Analysis

The prevalences of HR and LR HPV types by cytological class are reported. The proportion of HPV 16-18 and HPV 6-11-16-18 by cytological class are reported, including and excluding cases with co-infection of other HPV types. All analyses were performed separately for women under 35.

Co-infections of vaccine types with other types not included in vaccines are classified as follows: for HR vaccine types, HPV 16-18 alone (16, 18 or 16+18), HPV 16-18 + HR (HPV 16-18 + any non-vaccine high risk type independently from the presence of LR types), HPV 16-18 + LR (HPV 16-18 + any LR types, but without any co-infection of non-vaccine HR). Similarly, for LR vaccine types, HPV 6-11 alone (6, 11 or 6+11), HPV 6-11 + HR (HPV 6-11 + any non-vaccine high risk type, i.e. excluding 16-18, independently from the presence of other LR types), HPV 6-11 + LR (HPV 6-11 + any LR types, excluding any co-infection with HR types).

We estimated the age-adjusted Population Attributable Risk of Cytology abnormalities due to HPV vaccine type using a logistic model, according to the algorithm proposed by Fleiss [11], while 95% confidence intervals were computed according to the algorithm proposed by Rothman [12].

The analysis was performed taking into account the two stage sample using STATA 8 survey module [13].

### Ethics

The study was approved by the Ethics Committee of the Istituto Superiore di Sanità (the Italian National Institute of Health, CE-ISS 07-162 e 07/163). Women were informed of an additional HPV test in the screening procedure and asked to sign a consent form.

## Results

Table 1 reports the distribution of cytology results by recruiting centre. The proportion of inadequate samples ranges from 0 to 2.4%, while the proportion of ASC-US+ cytology ranges from 3.4% to 12.5%.

**Table 1:** Distribution of cytology results by recruiting centre

	Lazio		Campania		Abruzzo		Sicily		Sardinia		All	
	**N**	**%**	**N**	**%**	**N**	**%**	**N**	**%**	**N**	**%**	**N**	**%**

**Negative**	385	95.8	673	94.3	734	91.8	731	94.2	628	87.5	3151	92.4

**Positive**	14	3.5	24	3.4	48	6.0	31	4.0	90	12.5	210	6.2
***ASC-US***	*6*	*1.5*	*11*	*1.5*	*26*	*3.3*	*22*	*2.8*	*49*	*6.8*	*117*	*3.4*
***AGC***	*0*	*0.0*	*1*	*0.1*	*0*	*0.0*	*1*	*0.1*	*1*	*0.1*	*3*	*0.1*
***L-SIL***	*5*	*1.2*	*11*	*1.5*	*14*	*1.8*	*6*	*0.8*	*36*	*5.0*	*72*	*2.1*
***ASC-H***	*0*	*0.0*	*0*	*0.0*	*7*	*0.9*	*0*	*0.0*	*3*	*0.4*	*10*	*0.3*
***H-SIL***	*3*	*0.7*	*1*	*0.1*	*1*	*0.1*	*2*	*0.3*	*1*	*0.1*	*8*	*0.2*

**Inadequte**	3	0.7	17	2.4	18	2.3	14	1.8	0	0.0	52	1.5

**All**	402		714		800		776		718		3410	

Table 2 reports the HPV types found by cytology class. The overall positivity of HR types increases with cytology severity. HPV 16 is the most frequent virus strain in all classes, its frequency is higher in ASC-H and H-SIL (50% and 37%, respectively) than in ASC-US and L-SIL (7% and 22%, respectively). HPV 18 is rare in all classes (0.3% in normal, 0.4 in ASC-US, 4.2 in L-SIL and 0 in ASC-H and H-SIL). HPV 6 and 11 were not found in ASC-H and H-SIL and are present only in 3.5% and 5.6% of ASC-US and L-SIL samples, respectively.

**Table 2:** Distribution HPV types by cytology class

	**Negative**		ASC-US*		**L-SIL**		**ASC-H**		**H-SIL**		**Inadequate**		**All**	
							
	**N**	**%**	**N**	**%**	**N**	**%**	**N**	**%**	**N**	**%**	**N**	**%**	**N**	**%**
							
	3151		117	100.0	72	100.0	10	100.0	8	100	52	100	3410	100
**age**														
**25-34**	690	21.9	36	30.8	37	51.4	3	30.0	1	12.5	18	34.6	785	23.0
**35-44**	1071	34.0	50	42.7	21	29.2	3	30.0	4	50.0	15	28.8	1164	34.1
**45-54**	962	30.5	26	22.2	13	18.1	4	40.0	2	25.0	16	30.8	1023	30.0
**55-64**	428	13.6	5	4.3	1	1.4	0	0.0	1	12.5	3	5.8	438	12.8
														
**Hybrid capture**														
**HR+ only**	195	6.2	24	20.5	30	41.7	6	60.0	5	62.5	8	15.4	268	7.9
**LR+ only**	85	2.7	6	5.1	4	5.6	0	0.0	0	0.0	2	3.8	97	2.8
**HR & LR+**	27	0.9	10	8.5	19	26.4	0	0.0	1	12.5	0	0.0	57	1.7
**Total**	307	9.7	40	34.2	53	73.6	6	60.0	6	75.0	10	19.2	422	12.4
														
**Types**														
**6**	19	0.6	3	2.6	2	2.8	0	0.0	0	0.0	0	0.0	24	0.7
**11**	6	0.2	1	0.9	2	2.8	0	0.0	0	0.0	0	0.0	9	0.3
**16**	67	2.1	9	7.7	16	22.2	5	50.0	3	37.5	2	3.8	100	2.9
**18**	11	0.3	0	0.0	3	4.2	0	0.0	0	0.0	1	1.9	14	0.4
**26**	1	0.0	0	0.0	1	1.4	0	0.0	0	0.0	0	0.0	2	0.1
**31**	26	0.8	2	1.7	6	8.3	0	0.0	1	12.5	0	0.0	35	1.0
**33**	12	0.4	1	0.9	4	5.6	0	0.0	0	0.0	0	0.0	17	0.5
**35**	10	0.3	3	2.6	1	1.4	0	0.0	2	25.0	1	1.9	16	0.5
**39**	10	0.3	4	3.4	1	1.4	0	0.0	0	0.0	1	1.9	15	0.4
**40**	4	0.1	0	0.0	0	0.0	0	0.0	0	0.0	0	0.0	4	0.1
**42**	7	0.2	1	0.9	1	1.4	0	0.0	0	0.0	0	0.0	9	0.3
**43**	3	0.1	2	1.7	0	0.0	0	0.0	0	0.0	0	0.0	5	0.1
**44**	0	0.0	1	0.9	0	0.0	0	0.0	0	0.0	0	0.0	1	0.0
**45**	11	0.3	2	1.7	0	0.0	0	0.0	0	0.0	0	0.0	13	0.4
**51**	18	0.6	2	1.7	9	12.5	0	0.0	0	0.0	1	1.9	29	0.9
**52**	15	0.5	2	1.7	2	2.8	0	0.0	0	0.0	1	1.9	19	0.6
**53**	9	0.3	3	2.6	7	9.7	0	0.0	0	0.0	1	1.9	19	0.6
**54**	2	0.1	0	0.0	0	0.0	0	0.0	0	0.0	0	0.0	2	0.1
**56**	14	0.4	2	1.7	6	8.3	0	0.0	0	0.0	0	0.0	22	0.6
**58**	14	0.4	2	1.7	2	2.8	0	0.0	1	12.5	0	0.0	19	0.6
**59**	12	0.4	1	0.9	1	1.4	0	0.0	0	0.0	0	0.0	14	0.4
**66**	15	0.5	2	1.7	6	8.3	0	0.0	1	12.5	0	0.0	24	0.7
**68**	9	0.3	0	0.0	2	2.8	0	0.0	0	0.0	0	0.0	11	0.3
**70**	4	0.1	1	0.9	0	0.0	0	0.0	0	0.0	0	0.0	5	0.1
**73**	5	0.2	0	0.0	0	0.0	1	10.0	0	0.0	0	0.0	6	0.2
**81**	3	0.1	0	0.0	0	0.0	0	0.0	0	0.0	0	0.0	3	0.1
**82**	2	0.1	1	0.9	1	1.4	0	0.0	0	0.0	0	0.0	4	0.1
														
**co-infections HR+**														
**2**	53	1.7	8	6.8	16	22.2	0	0.0	2	25.0	0	0.0	79	2.3
**3**	13	0.4	1	0.9	3	4.2	0	0.0	0	0.0	0	0.0	17	0.5
**4**	1	0.0	0	0.0	1	1.4	0	0.0	0	0.0	0	0.0	2	0.1

HR: high risk; LR: low risk (7)

* Including 3 Atypical Glandular Cells (AGC), only one positive for HPV 16.

Prevalence of HR infections is double in women with inadequate cytology as compared to that observed in women with negative cytology.

Table 3 shows the fraction of cytologic abnormalities in the population attributable to HPV 16-18 and HPV 6-11 infections alone, in co-infection with other HR types or in co-infections with only LR types. The sum of fraction attributable to HPV 16-18 alone and in co-infection with LR types is 12.1% and 20.6% for ASC-US+ and L-SIL+, respectively. The proportion rises to 15.4% and 28% for ASC-US+ and L-SIL+, respectively, if we include also co-infections with other HR types. The inclusion of HPV6 and 11 adds 0.7% to the attributable fraction of the ASC-US cytology and has no effect on the L-SIL+ cytology.

**Table 3:** Fraction of cytology abnormalities in the population attributable to HPV 16-18 and HPV 6-11 alone and in co-infection with high and low risk types (7).

ALL	PAP TEST RESULTS							
			
	Negative		ASC-US		L-SIL+			
	3151		117		90			
HPV INFECTION	N	%	N	%	N	%	PAR ASC-US+ (95% CI)	PAR L-SIL+ (95% CI)
HPV negative	2844	90.3	77	65.8	25	27.8		
16-18 alone	43	1.4	8	6.8	17	18.9	11.2 (6.6-15.7)	18.4 (9.8-26.1)
16-18 & other HR+	28	0.9	1	0.9	7	7.8	3.3 (0.5-6.0)	7.4 (1.7-12.8)
16-18 & LR+	3	0.1	0	0.0	2	2.2	0.9 (0.0-2.2)	2.2 (0.0-5.2)
6-11 alone	17	0.5	1	0.9	0	0.0	0.2 (0.0-1.1)	
6-11 & HR+ (excluded 16-18)	2	0.1	1	0.9	2	2.2	1.4 (0.2-3.0)	2.2 (0.0-5.2)
6-11 & other LR+	1	0.0	1	0.9	0	0.0	0.5 (0.5-1.4)	
other HR+ only	142	4.5	22	18.8	32	35.6	23.4 (16.5-29.6)	33.9 (22.9-43.4)
other LR+ only	63	2.0	5	4.3	3	3.3	2.7 (0.0-5.5)	2.7 (0.0-6.4)
other HR+ & LR+	8	0.3	1	0.9	2	2.2	1.3 (0.0-2.9)	2.1 (0.0-5.1)
								
**< = 35 years**								
	**PAP TEST RESULTS**							
			
	**Negative**		**ASC-US**		**L-SIL+**			
	**771**		**42**		**41**			
**HPV INFECTION**	**N**	**%**	**N**	**%**	**N**	**%**	**PAR ASC-US+ (95% CI)**	**PAR L-SIL+ (95% CI)**

HPV negative	649	84.2	22	52.4	9	22.0		
16-18 alone	19	2.5	4	9.5	8	19.5	13.4 (5.3-20.8)	18.8 (5.6-30.2)
16-18 & other HR+	17	2.2	1	2.4	4	9.8	5.1 (0.0-10.2)	9.1 (0.0-17.9)
16-18 & LR+	1	0.1	0	0.0	1	2.4	1.2 (0.0-3.5)	2.4 (0.0-7.0)
6-11 alone	8	1.0	1	2.4	0	0.0	0.7 (0.0-3.1)	
6-11 & HR+(exclued 16-18)	2	0.3	0	0.0	2	4.9	2.3 (0.0-5.6)	4.8 (0.0-11.2)
6-11 & other LR+	1	0.1	1	2.4	0	0.0	1.2 (0.0-3.5)	
other HR+ only	52	6.7	9	21.4	15	36.6	26.0 (14.7-35.8)	34.8 (17.5-48.4)
other LR+ only	19	2.5	3	7.1	0	0.0	2.5 (0.0-6.7)	
other HR+ & LR+	3	0.4	1	2.4	2	4.9	3.5 (0.0-7.4)	4.8 (0.0-11.1)

All PAR adjusted by age.

In women younger than 35, the HPV16-18 attributable fraction is slightly higher, even if not significantly: 14.6% and 21.2% for ASC-US+ and L-SIL+, respectively including only LR co-infections; 19.7% and 30.3% for ASC-US+ and L-SIL+, respectively, if we also include co-infections with other HR types. The inclusion of HPV 6 and 11 adds 1.9% to the attributable fraction of the ASC-US cytology and has no effect on the L-SIL+ cytology.

## Discussion

### HPV prevalence

The overall HPV prevalence found in this study was similar to other Italian population-based studies [14,15]. A detailed discussion about type specific HPV prevalence in Central and Southern Italy based mostly on these data was presented elsewhere (3). Here, we report the results of HPV typing by cytology class, a piece of information that was not available at the time of the above mentioned publication.

While, as expected, the prevalence of HR infections increases with cytology severity, it is worth underlining that HR HPV prevalence is double in inadequate samples as compared to negative ones. A cost effectiveness analysis of a possible use of HPV DNA test [16] compared to repeated cytology should be performed, particularly taking into account the very low compliance with repeated cytology, i.e. about 60% [17].

The HPV prevalence in general population is quite low in Italy compared to other countries. More surprisingly, the observed low prevalence persists in all cytology classes, compared to other studies [18-20]. There are two possible explanations for the low prevalence of HPV infections in cytological abnormalities: 1) the specificity of cytology in southern Italy screening programs may be sub-optimal and consequently our data cannot be generalised to other screening programs in similar conditions; 2) the low prevalence of infections may contribute to a low positive predictive value of cytology for infection itself. The two hypotheses are not mutually exclusive. Whatever the reason, in Italy it could be useful to perform a triage test with HPV DNA not only for ASC-US+ samples but also for L-SIL in women over 35, as previously showed by Ronco and collaborators [21].

In all cytology classes HPV 16 is the most prevalent type and, overall, its prevalence is more than twice the prevalence of the type ranking second, HPV31. Nevertheless, the proportion of cytological abnormalities reasonably due to HPV 16 or 18 is negligible for ASC-US, moderate for L-SIL and relevant only for ASC-H and H-SIL, even if for these two last cytological classes our estimates are based on few observations and cannot be accurate. Given the relative frequency of cytology classes in the screening population, the overall impact on screening program positivity rate of a bivalent vaccine (16-18) would be 12-15% and that of a quadrivalent vaccine (6-11-16-18) would be 13-16%. A modest effect compared to the variability observed among different programs or to what would be obtained with a triage test strategy for ASC-US samples, still scarcely implemented in Italian screening programs.

Furthermore, we must take into account that reduction in prevalence will probably increase the proportion of false positives, i.e. a decrease of Pap-test Positive Predictive Value for low grade lesions [22]. Consequently, the decrease in ASC-US and L-SIL prevalence will not be translated into an equal decrease in screening programs positivity rate.

Actually, the targets of mass vaccination are pre-adolescent girls and screening programs will see the vaccinated cohorts when these girls are 25. Even if we consider only younger women, the impact of vaccine on cytology positivity would not be substantially different.

On the other hand, a more relevant impact on CIN2 and CIN3 reduction is expected: in a large study conducted in the same geographic area [23] more than 70% CIN2+ was due to HPV16-18. The final consequence of the differential impact on cytology and histology will be a dramatic decrease of cytology PPV. This scenario has already been hypothesized by other authors [24-26]. Our data bring another small piece of evidence from Italian screening programs, confirming its likelihood.

The shift to HPV DNA as primary screening test may change the scenario [25,26], but at the moment in younger women the results about screening with HPV test are contradictory [27]. Furthermore the implementation of a completely new screening algorithm will be a gradual process. Consequently, we cannot exclude that the first vaccinated cohorts will be still screened with Pap-test.

### Limits

We tested women who were invited and participated to cervical cancer screening programs in Rome and southern Italy. Response to organised screening is quite low, ranging from 25 to 50% in the selected areas; low participation may introduce a self-selection bias. Nevertheless, active invitation, for the study or for the screening, is the only mean we had of reducing self-selection bias in surveys. Previous studies found few socio-economic differences between participants and not participants [28,29].

A second limit, due to the nesting of this study into organised screening programs, is that we could not include some regions, such as Calabria and Apulia, because at the time the study started there were no active programs contacting the whole target population.

We typed only women positive to HR and LR HCII probes; according to this protocol we may have missed women who are positive to other HPV types not included in the probes or samples with very low number of virus DNA copies. This typing strategy was the same used in all the pre-vaccination studies sponsored by the Ministry of Health making the results comparable [14,15] and in many international studies included in the largest meta-analysis ever published to date [30].

In this report, the vaccine cross-protection against HPV strains not present in the vaccine mix was not taken into consideration. On this issue, only rough estimates can be put forward. Moreover, different definitions of cross-protection values are reported: for quadrivalent vaccines, cross-protection refers to HPV 31, 33, 45, 52, 58 infection and was estimated to be 25% [31], while for bivalent vaccines it refers to all non-vaccine HR types persistent infection (at least for 6 months) and was shown to be 11% [32]. According to these data, the reduction values reported here would be increased by 2.3% (25%*9.2%, i.e. the PAR for 31, 33, 45, 52, 58 types) and 2.5% (11%*23%, i.e. the PAR for non-vaccine HR types), respectively.

## Conclusions

The impact of mass vaccination on cytology screening programs recall rate would be negligible, while probably its impact on CIN2+ detection rate would be much stronger. Our data support the hypothesis that the positive predictive value of Pap test in vaccinated women would be dramatically lower than in non-vaccinated women, as argued by several authors.

## Abbreviations

AGC: Atypical Glandular Cellls; ASC-H: Atypical Squamous Cells non excluding High Grade lesions; ASC-US: Atypical Squamous Cells of Undetermined Significance; ASC-US+: Atypical Squamous Cells of Undetermined Significance or more severe cytology, it includes ASC-US, L-SIL, ASC-H, H-SIL and cancer; HCII: Hybrid Capture II^®^; HPV: human papillomavirus; HR HPV: high risk human papillomavirus types; H-SIL: High Grade Squamous Intraepithelial Lesion; LR HPV: low risk human papillomavirus types; L-SIL: Low Grade Squamous Intraepithelial Lesion; L-SIL+: Low Grade Squamous Intraepithelial Lesion or more severe, it includes L-SIL, ASC-H, H-SIL and cancer; NTCC: New Technologies in Cervical Cancer; PAR: Population Attributable Risk; PCR: polymerase chain reaction; RLU/CO: Relative light units/cut-off; STM: standard transport medium

## Competing interests

Financial: PGR received travel reimbursement for two international conferences by Sanofi Pasteur MSD; FMC is occasional advisor to Gen-Probe, Abbot, Sanofi and GlaxoSmithKline.

Non financial: PGR is employed in the HTA Unit of the Agency for Public Health of the Lazio Region, this Agency contributes in establishing public screening programs.

## Authors' contributions

PGR designed and conducted the study and drafted the paper; FCa designed the laboratory procedures, coordinated the laboratory quality control and contributed in drafting the paper; SB and EB performed the typing test; FCh designed the data flow, managed the data and performed the statistical analyses; AS, AL, RM, MTP, ADI coordinated the study in the recruiting centres. The Working Group contributed in recruiting the women, performing the HPV tests, collecting the data.

All the authors approved the final version of the paper.
